# Supplementary material for: Predicting ADC Map Quality from T2-Weighted MRI: A Deep Learning Approach for Early Quality Assessment to Assist Point-of-Care
Source: medRxiv. 2025 Jan 15:2025.01.15.25320592. Preprint. [Version 1] doi: 10.1101/2025.01.15.25320592 (PMC12191087; doi:10.1101/2025.01.15.25320592)
Supplement: 1 [file NIHPP2025.01.15.25320592V1-supplement-1.pdf]

medRxiv preprint doi: https://doi.org/10.1101/2025.01.15.25320592; this version posted January 15, 2025. The copyright holder for this preprint (which was not certified by peer review) is the author/funder, who has granted medRxiv a license to display the preprint in perpetuity. This article is a US Government work. It is not subject to copyright under 17 USC 105 and is also made available for use under a CC0 license.

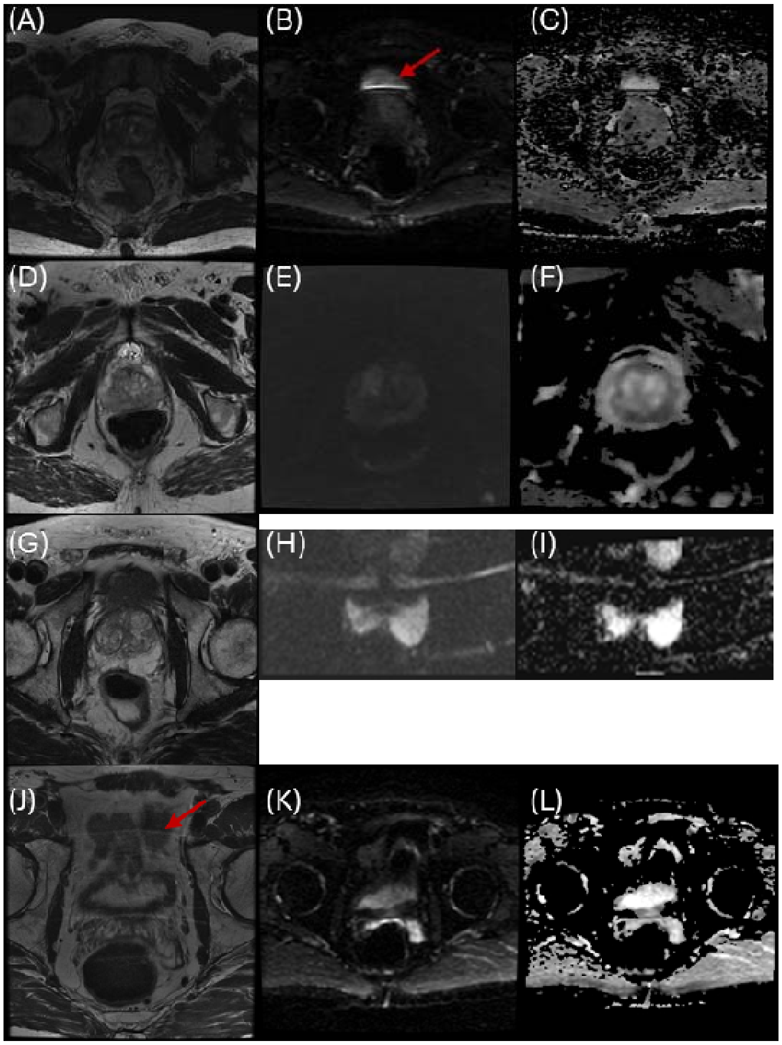

Figure S1: Representative examples of common image quality issues in prostate MRI. **(A-C)** Susceptibility artifacts due to rectal gas: T2-weighted image showing subtle geometric distortion **(A)**, corresponding high b-value DWI with signal pileup at the prostate-rectum interface **(B, arrow)**, and ADC map demonstrating signal dropout and reduced contrast **(C)**. **(D-F)** Low contrast artifacts: T2-weighted image with normal anatomy **(D)**, DWI showing poor zone differentiation **(E)**, and corresponding low-contrast ADC map **(F)**. **(G-I)** Aliasing artifacts from reduced field of view: Normal T2-weighted image with expanded FOV **(G)**, reduced FOV DWI with bright band artifacts **(H)**, and ADC map showing wraparound effects **(I)**. **(J-L)** Motion artifact apparent in the T2 image **(J, arrow)**

medRxiv preprint doi: https://doi.org/10.1101/2025.01.15.25320592; this version posted January 15, 2025. The copyright holder for this preprint (which was not certified by peer review) is the author/funder, who has granted medRxiv a license to display the preprint in perpetuity.
This article is a US Government work. It is not subject to copyright under 17 USC 105 and is also made available for use under a CC0 license.

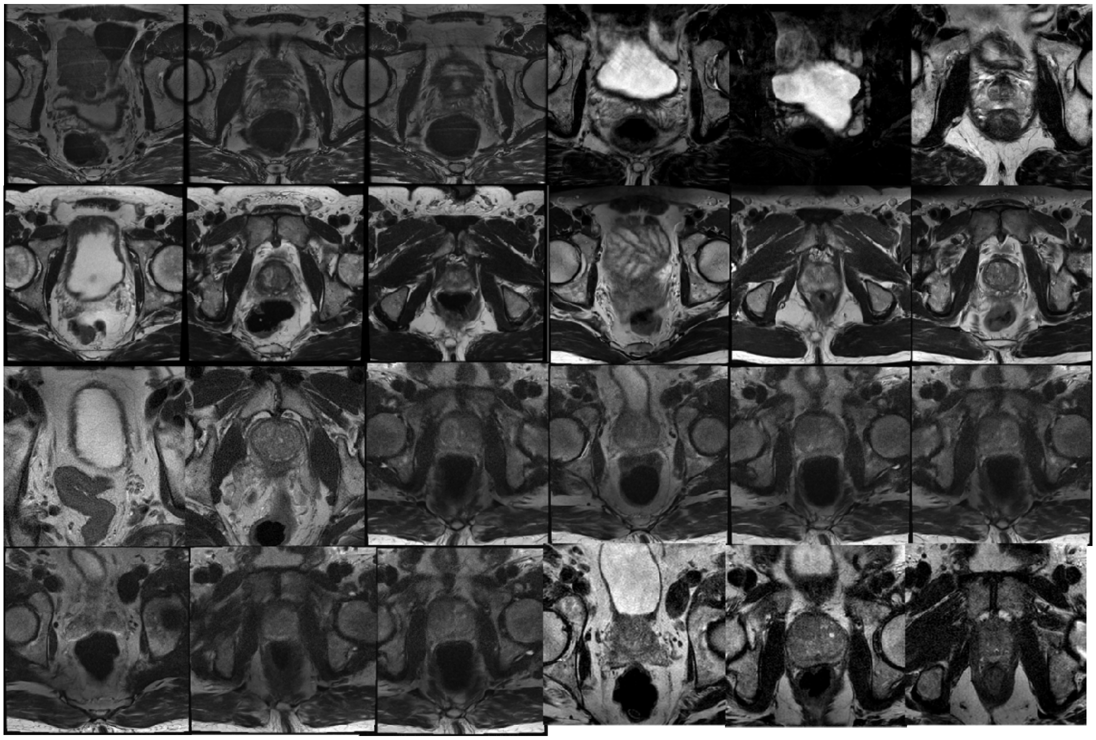

Figure S2: Representative T2-weighted images from cases with non-diagnostic quality at both in-house (left 3 images) and external sites (right). Each row shows consecutive axial slices through the prostate demonstrating how bladder distension, often associated with benign prostatic hyperplasia, compromises image quality. Note the distorted anatomy and signal inhomogeneity across multiple slices, making these studies clinically uninterpretable

medRxiv preprint doi: https://doi.org/10.1101/2025.01.15.25320592; this version posted January 15, 2025. The copyright holder for this preprint (which was not certified by peer review) is the author/funder, who has granted medRxiv a license to display the preprint in perpetuity.
This article is a US Government work. It is not subject to copyright under 17 USC 105 and is also made available for use under a CC0 license.

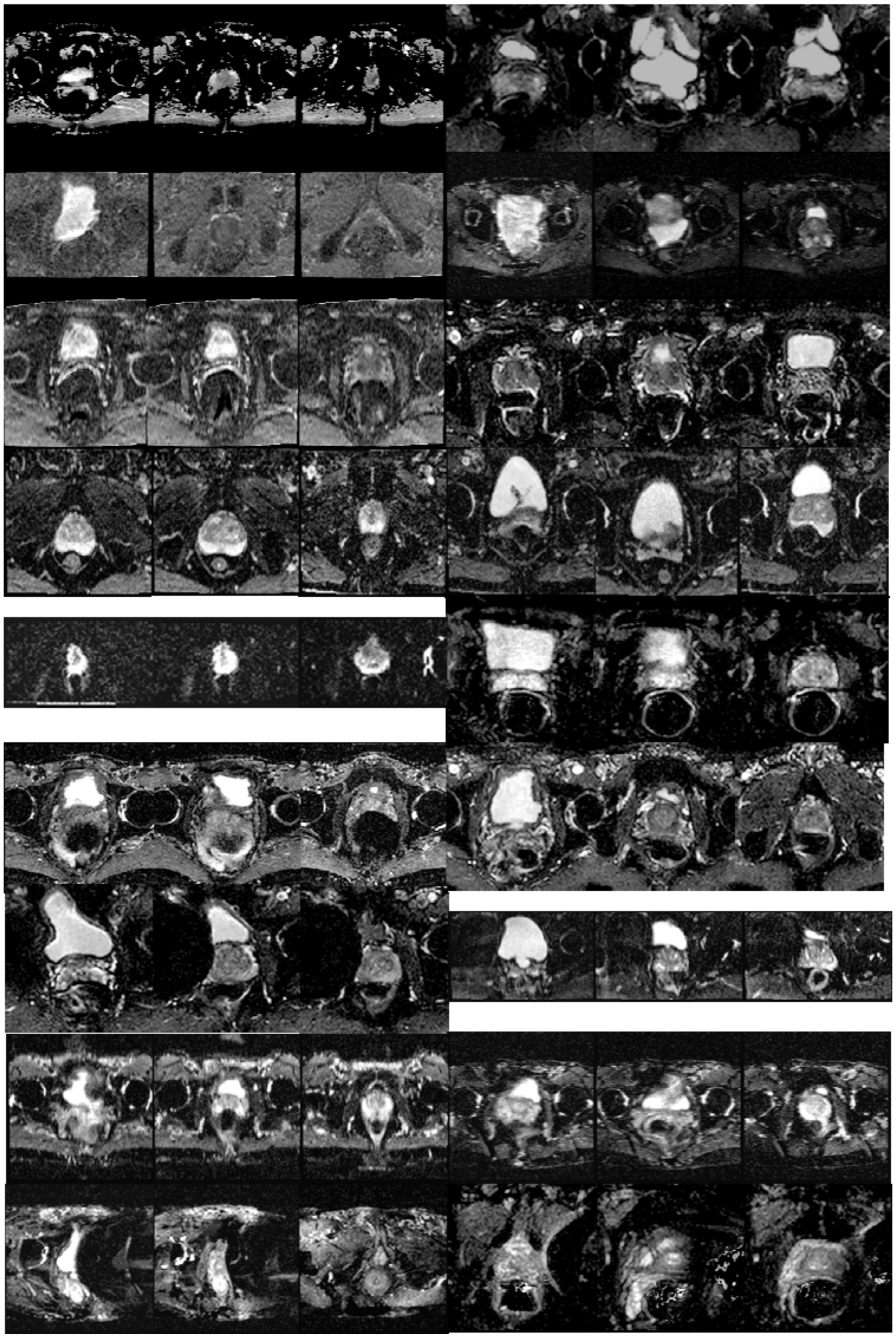

Figure S3: Representative ADC maps from cases with non-diagnostic quality at both in-house and external sites. Each row shows consecutive axial slices through the prostate, illustrating how bladder distension, often in conjunction with benign prostatic hyperplasia, leads to significant image degradation. Note the susceptibility artifacts and geometric distortions across multiple slices that render these studies non-diagnostic.
